# Supplementary material for: Observation of magnetic skyrmion lattice in Cr0.82Mn0.18Ge by small-angle neutron scattering
Source: Sci Rep. 2025 Jan 22;15:2865. doi: 10.1038/s41598-025-86652-1 (PMC11754481; doi:10.1038/s41598-025-86652-1)
Supplement: Supplementary file 1 — Supplementary Information. [file 41598_2025_86652_MOESM1_ESM.pdf]

Supplementary information for ”Observation of magnetic skyrmion lattice in $Cr_{0.82}Mn_{0.18}Ge$ by small-angle neutron scattering”

Victor Ukleev[1,*], Tapas Samanta[2], Oleg I. Utesov[3], Jonathan S. White[4], and Luana Caron[2,1]

[1]Helmholtz-Zentrum Berlin für Materialien und Energie, D-13109 Berlin, Germany
[2]Department of Physics, Bielefeld University, Bielefeld 33501, Germany
[3]Center for Theoretical Physics of Complex Systems, Institute for Basic Science, Daejeon 34126, Republic of Korea
[4]Laboratory for Neutron Scattering and Imaging (LNS), PSI Center for Neutron and Muon Sciences, Paul Scherrer Institute, CH-5232 Villigen PSI, Switzerland
[*]victor.ukleev@helmholtz-berlin.de

Topological Hall Effect

Figure S1 shows bulk characterization of the $Cr_{0.82}Mn_{0.18}Ge$ sample that allows to extract the topological contribution to Hall effect (THE). Fig. S1a shows magnetization curves of $Cr_{0.82}Mn_{0.18}Ge$ near the ordering temperature that reveal typical to helimagnets behavior similar to MnSi[1]. Figure S1b demonstrates the magnetic field dependence of magnetoresistance (MR) which also shows the gradual behavior. These results are consistent with the previous study[2]. Figure S1c shows the magnetic field dependencies of the Hall resistivity (ρ_{xy}). In order to isolate the topological Hall contribution to ρ_{xy}, the ordinary and anomalous Hall components were subtracted from the data (Fig. S1d). The dip in THE corresponds well to the stability window of the $A-$phase where skyrmions are also observed by means of SANS. The emergent magnetic field due to a skyrmion is estimated by the relation $B_{\rm em} = -(h/e)/L_s^2$, where L_s is the magnetic period of the skyrmion lattice (SkL). The magnitude of $B_{\rm em} \approx 2.6\,\mathrm{T}$ is of the same order as in MnSi[3].

Magnetic phase boundaries

Figure S2 presents the SANS intensity dependence on the magnetic field for the sector boxes corresponding to the conical (Figs. S2a–c) and SkL (Figs. S2d–f) peaks at various temperatures. This data was used to construct the two-dimensional magnetic phase diagrams shown in Figure 2 of the main text. The finite intensity observed in the boxes corresponding to the conical peaks around the A-phase (Figs. S2b, c) suggests phase coexistence with skyrmions.

References

1. Lee, M., Onose, Y., Tokura, Y. & Ong, N. Hidden constant in the anomalous hall effect of high-purity magnet MnSi. *Physical Review B—Condensed Matter and Materials Physics* **75**, 172403 (2007).

2. Zeng, H. *et al.* Low-field induced topological hall effect in chiral cubic $Cr_{0.82}Mn_{0.18}Ge$ alloy. *Journal of Alloys and Compounds* **868**, 159057 (2021).

3. Neubauer, A. *et al.* Topological Hall effect in the A phase of MnSi. *Physical Review Letters* **102**, 186602 (2009).

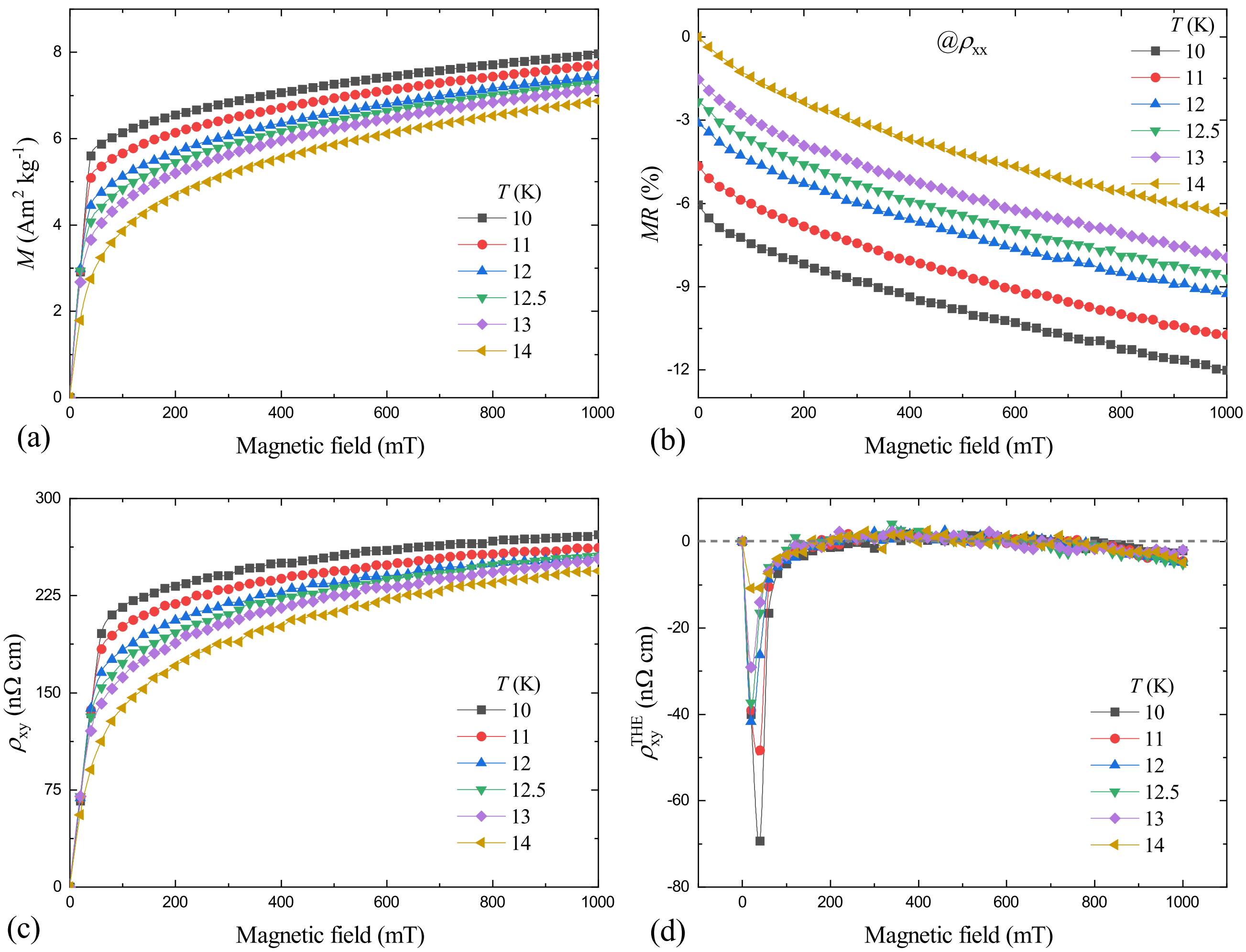

Figure 1. $Cr_{0.82}Mn_{0.18}Ge$ sample characterization by means of (a) magnetization measurement, (b) magnetoresistance, and (c) Hall effect. The extracted topological Hall signal (d) corresponds to the formation of the skyrmion lattice in the A−phase.

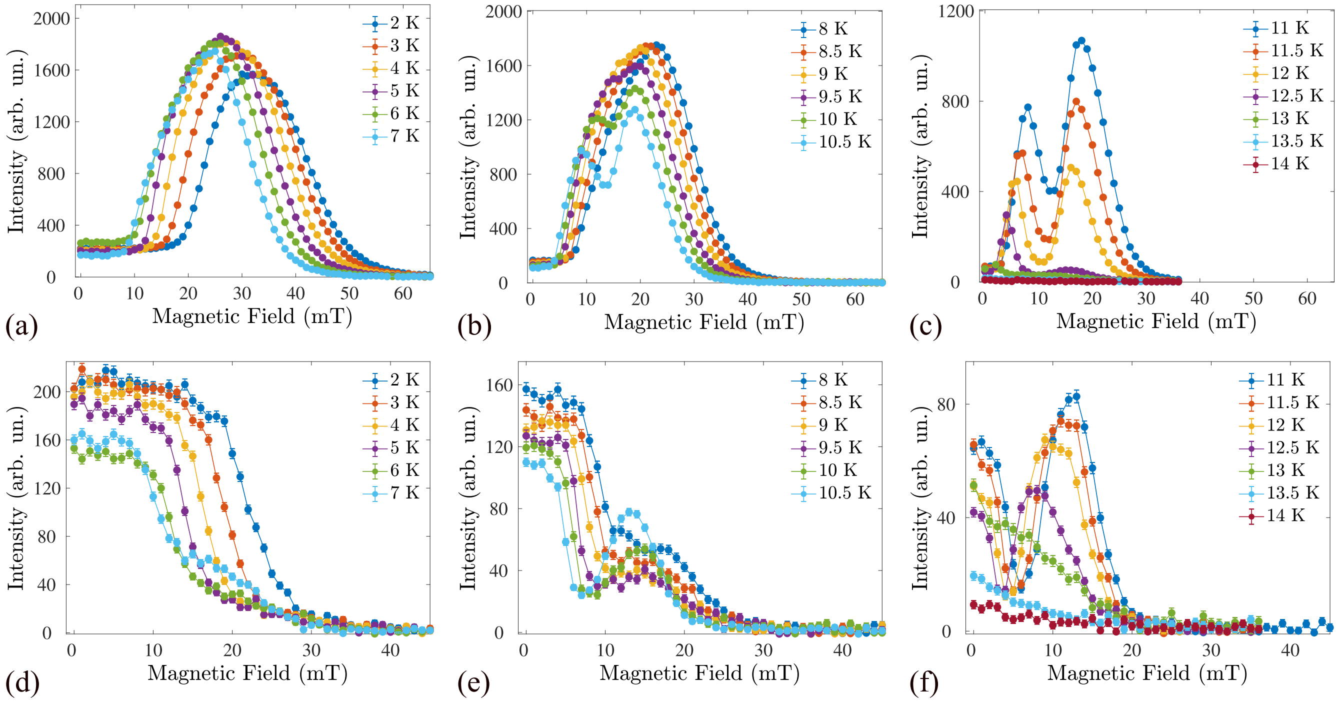

Figure 2. Magnetic field dependence of the SANS peak intensities for conical (a–c) and SkL (d–f) phases at different temperatures.
